# Supplementary material for: WordCommentsAnalyzer: A windows software tool for qualitative research
Source: F1000Res. 2018 Sep 4;7:536. Originally published 2018 May 3. [Version 2] doi: 10.12688/f1000research.14819.2 (PMC6137411; doi:10.12688/f1000research.14819.2)
Supplement: Supplementary file 3 [file f1000research-7-17614-s0002.tgz › b2a79496-4ada-47c4-9682-a99ce867836d.zip!/Eight Iranian Health Journals/Journal of Knowledge and Health_34.docx]

© Journal of Knowledge and Health. This work is licensed under CC BY 4.0 (https://creativecommons.org/licenses/by/4.0/).

Volume & Issue: دوره 4، شماره 4: 1388

Title: ميزان جابجايي و چرخش مهره¬هاي کمری در رادیوگرافی فلکسیون- اکستانسیون

http://knh.shmu.ac.ir/index.php/site/article/view/188

مقدمه: با توجه به عدم وجود اطلاعات كافي در خصوص ميزان جابجايي و چرخش مهره­هاي كمري در جامعه ايراني از اين­رو مطالعه حاضر به بررسی ميزان جابجايي و چرخش مهره­هاي کمری مردان سالم در جامعه ایران مي­پردازد. مواد و روش­ها: در این مطالعه توصیفی، 22 مرد داوطلب سالم در محدوده سنی 43-21 سال شرکت داشتند. متغیرهای رادیوگرافی شامل جابجایی و چرخش مهره­ها در سطح ساجیتال بودند که به روش پنجابي محاسبه شدند. از روش پوتو برای اخذ رادیوگرافی فلکسیون- اکستانسیون استفاده شد. سه کلیشه در وضعیت­های طبيعي، فلکسیون و اکستانسیون کامل از بیماران اخذ شد. پس از اسکن، کلیشه­ها وارد محیط نرم­افزار CARA شده و متغیرهای مربوطه از آن استخراج شدند. از آماره­های توصیفی به­منظور توصیف متغیرهای مورد مطالعه استفاده شده است نتايج: جابجايي 15/1 ميلي­متر در سطح L1-2، 58/1 ميلي­متر در سطح L2-3، 44/2 ميلي­متر سطح L3-4، 80/2 ميلي­متر در سطح L4-5 و 18/1 ميلي­متر در سطح L5-S1 به­دست آمد. ميزان چرخش 25/10 درجه در سطح L1-2، 83/11 درجه در سطح L2-3، 61/13 درجه در سطح L3-4، 11/17 درجه در سطح L4-5 و 26/16 درجه در سطح L5-S1 حاصل شد. نتيجه­گيري: نتایج حاصل از مطالعه حاضر نشان­دهنده متوسط جابجایی کم­تر از 3 میلی­متر و متوسط چرخش کم­تر از 20 درجه در سطوح حرکتی ستون فقرات کمری می­باشد كه موافق با نظر برخي پژوهشگران و مخالف برخي ديگر است.

Title: بررسي ارتباط بين نسبت اندازه¬هاي سر و گردن فمور به اندازه¬هاي ميله (Nail) در پيامد درمان شكستگي¬هاي اينتروتروكانتريك به روش Dynamic Hip Screw

http://knh.shmu.ac.ir/index.php/site/article/view/189

مقدمه: درمان شکستگی اینترتروکانتریک امروزه یکی از معضلات اصلی در سنین بالا محسوب می­شود. به­خصوص در شکستگی­های ناپایدار و بیماران دارای استئوپروز شدید نتایج درمان جراحی مطلوب نمی­باشد. عوامل مختلفی در نتایج درمان مؤثرند که در این مطالعه یک معیار جدید که احتمالاً در پیش آگهی درمان مؤثر است، مطرح گردیده است. مواد و روش­ها: از مهر سال 1382 تا شهریور 1386، تعداد 128 بیمار که شکستگی اینترتروکانتریک ناپایدار داشته يا از نظر معیار (سايز) sighs استئوپروز نسبتاً شدید داشته­اند (گروه 3- 1) و به روش DHS عمل جراحی شده­اند و در تمام بیماران فاصله نوك تا انتها (Tip Apex Distance) در محدوده mm25 بوده است، مطالعه شده­اند. در این بیماران معیار Y/B (نسبت قطر گردن فمور در میانه آن به قطر بدنه (Nail)) X/A (نسبت بیش­ترین قطر سر فمور به قطر سر ميله) اندازه­گیری شده و میزان شکست درمان در گروه­های مختلف اندازه­گیری شده است. نتایج: میزان کلی شکست درمان 26% (33 مورد) بوده، میانگین سنی بیماران 4/74 سال، 86 بیمار زن و 42 بیمار مرد بوده­اند. بیش­ترین میزان شکست درمان (21 مورد) در گروهB1  که معيار mm 6/3 ≤Y/B و هم­چنین در گروه 2B (23 مورد) که mm 3≤X/A داشته­اند، دیده شده است. میانگین معیار Y/B، 64/3 ميلي­متر و میانگین معیار X/A، 08/3 ميلي­متر بوده است. نتيجه­گيري: باتوجه به میزان بالای شکست درمان در کل بیماران (26%) و به­ویژه میزان بالای آن در گروه­های 1 Bو 2 Bکه نشان­دهنده بالا بودن شکست درمان در اندازه­هاي بالای سر و گردن فمور می­باشد، باید در بیماران با شکستگی ناپایدار به­ویژه افراد دارای اندازه­هاي بالای سر و گردن فمور تمهیدات جدیدی برای بهبود پیش آگهی در نظر گرفته شود.

Title: بررسي اثر تزريق مهاركننده آدنيليل سيكلاز (SQ-22536) بر پردردی ناشی از تسهیل سیناپسی منطقه سري شكمي- مياني بصل¬النخاع موش صحرايي

http://knh.shmu.ac.ir/index.php/site/article/view/190

مقدمه: فعال شدن مسير آدنوزین مونوفسفات حلقوی (cAMP) سبب پردردي مکانيکي، حساس شدن نورون­هاي دردي مسير نخاعي و فسفريله شدن مسیر مولکولی پرتئین باندشونده به جزء پاسخ­دهنده به cAMP CREB)) مي­شود که نسخه­برداري ژني را شروع مي­کند. بنابراين در اين مطالعه نقش آدنيليل سيكلاز و مسير cAMP در تسهيل سيناپسي در منطقه سري شكمي- مياني بصل­النخاع (RVM) به­دنبال تزريق فرمالين در كف پاي حيوان مورد بررسي قرار گرفت. مواد و روش­ها: در این مطالعه از 36 سر موش صحرایی نژاد اسپراگ- دالی در محدوده­ی وزنی 300-250 گرم استفاده گردید. حیوانات به­طور تصادفی به 6 گروه شاهد، حلال دارو (سالین)، شاهد جراحی و مقادیر 50، 100 و 200 نانومول SQ-22536 تقسیم شده و فرمالین در پای راست حیوانات تزریق گردید. ميانگين نمره رفتارهاي درد با استفاده از آزمون Anova در گروه­هاي مختلف مقايسه شده است. نتایج: تزريق داخلRVM  غلظت 100 نانومولار SQ-22536 توانست در دقايق 5، 10، 15، 50 و 55 علایم رفتاري درد ناشي از تزريق فرمالين را به­طور معناداري (05/0>P) کاهش دهد. هم­چنين غلظت 200 نانومولار SQ-22536 تنها در دقيقه 15 رفتار دردي را کاهش داد. نتیجه­گیری: بنابراين مهار آدنيليل سيكلاز به­وسيله SQ-22536 داخل  RVMسبب کاهش رفتارهاي دردي ناشي از آزمون فرمالين در موش صحرايي مي­شود.

Title: بررسي اثر بخشي كرم 5% تري كلرواستيك اسيد در درمان ضايعات ليشمانيوز پوستي

http://knh.shmu.ac.ir/index.php/site/article/view/191

مقدمه: بيماري ليشمانيوز جلدي (سالك) يك بيماري اندميك و گسترده در سطح ايران مي­باشد. اسكار نازيبايي كه از اين بيماري به­جا مي­ماند به­عنوان يك مشكل جدي رواني براي اين بيماري قلمداد مي­شود. اين مطالعه جهت بررسي اثر كرم 5% تري كلرواستيك اسيد در درمان ضايعات ندولر پاپولر ليشمانيوز جلدي طراحی گردید. مواد و روش­ها: در مطالعه case series از بيماران مبتلا به سالك مراجعه­كننده به مركز تحقيقات بيماري­هاي پوستي و سالك، اسمير به­عمل آمد و در صورت مثبت بودن اسمير به­صورت تصادفي تحت درمان موضعي كرم TCA 5% روزانه 2 بار تا 8 هفته یا تا زمان بهبودي (اپیتلیزاسیون) كامل ضايعه قرار گرفتند. پس از پايان دوره درمان، بيماران بر اساس نحوه پاسخ به درمان در سه گروه بهبودي كامل، بهبودي نسبي و عدم بهبودي قرار گرفتند. بيماران 6 ماه پس از بهبودي كامل ضايعه از نظر اندازه اسكار ناشي از اين بيماري بررسي شدند. يافته­هاي به­دست آمده توسط نرم­افزار SPSS وارد رايانه شده و مورد تجزيه و تحليل قرار گرفت. نتايج: ميانگين مساحت ضايعه قبل از درمان mm290/81± 81/38 بود كه پس از 6 ماه از اتمام درمان mm21/9± 6/3 شده است. میزان بهبودي كامل از 1 نفر (3/6%) در هفته دوم به 13 نفر (%7/86) در هفته هفتم رسيده و در هفته هشتم كليه بيماران پاسخ كامل داده بودند. بيماران عارضه مهمی پيدا نكردند و فقط يك بيمار دچار اريتم مختصر در هفته دوم و ادم در ناحيه مصرف كرم در هفته پنجم پيدا كرد كه پس از 2 هفته برطرف شد. نتيجه­گيري: با توجه به حصول بهبودي كامل در كليه بيماران اين مطالعه در هفته هشتم، عوارض و هزينه كم دارو نسبت به درمان­هاي مشابه و اثربخشي تري كلرواستيك اسيد در بهبودي اسكار، به­نظر مي­رسد استفاده از كرم TCA 5% مي­تواند به­عنوان يك درمان جايگزين در بيماران مبتلا به سالك مطرح شود. طراحی یک مطالعه دو سویه کور در این راستا جهت حصول نتیجه­گیری قابل اعتماد ضروری می­باشد.

Title: مقایسه سه آزمون عملکردی تعادل در شناسایی سالمندان با و بدون سابقه زمین خوردن

http://knh.shmu.ac.ir/index.php/site/article/view/192

مقدمه: ارزیابی تعادل برای بیش­تر سالمندان در زمینه سلامت و بهداشت به­منظور کمک در جهت اهداف درمانی مناسب، افزایش آگاهی از خطر زمین خوردن و تخصیص وسیله کمکی مناسب لازم و ضروری است. زمین خوردن ارتباط مستقیمی با سلامت عمومی افراد سالمند دارد و شناسایی زود هنگامش باعث کاهش این خطر می‌شود. بنابراین پژوهش حاضر با هدف مقایسه سه روش ارزیابی عملکردی در شناسایی سالمندان با خطر زمین خوردن و بدون آن صورت گرفته است. مواد و روش‌ها: تحقیق حاضر از نوع علی- مقایسه‌ای بود که بدین منظور تعداد 52 سالمند مرد و زن از سه پارک شهر تهران بعد از تکمیل پرسش­نامه ارزیابی هوشیاری به­صورت در دسترس انتخاب و بنابر گزارش خود افراد، به دو گروه سالمند با سابقه زمين خوردن (27 نفر) و بدون سابقه زمین خوردن (25 نفر) تقسیم شدند. داده‌ها با استفاده از آزمون‌های: تعادل برگ، دسترسی عملکردی و آزمون زماندار برخاستن و برگشتن جمع­آوری شدند. تجزیه و تحلیل داده‌ها با استفاده از تحلیل واریانس چند متغیری (MANOVA) و ضریب همبستگی پیرسون در سطح معنا‌داری 05/0>P انجام شد. نتایج: نتایج نشان داد که تفاوت معناداری بین گروه­های سالمندان با سابقه زمین خوردن و بدون آن، در متغیر وابسته خطر زمین خوردن وجود دارد (0005/0=P) به­طوری­که سالمندان بدون سابقه زمین خوردن در همه آزمون­ها وضعیت بهتری داشتند. هم­چنین نشان داده شد که هر سه آزمون نسبت به هم دارای همبستگی معناداری هستند. نتیجه­گیری: به­طور کلی می‌توان گفت هر سه آزمون در دو گروه دارای تفاوت­هاي معناداری هستند و به کمک این ابزار می­توان اختلال تعادل سالمندان را شناسایی کرد.

Title: بررسی ارتباط کمردرد با عوامل روانی- اجتماعی و استرس های روانشناختی شغلی در پرستاران بیمارستان¬های شهر آمل

http://knh.shmu.ac.ir/index.php/site/article/view/193

مقدمه: پرستاران به­عنوان یک گروه پرخطر برای ابتلا به کمردرد شغلی شناخته شده­اند. این پژوهش به­منظور تعیین ارتباط کمردرد با عوامل روانی- اجتماعی و استرس­های روانشناختی شغلی در پرستاران بیمارستان­های شهر آمل انجام شد. مواد و روش­ها: در این مطالعه تحلیلی 400 نفر از پرستاران شاغل در بیمارستان­های شهر آمل پرسش­نامه­ای شامل 3 قسمت اطلاعات دموگرافیک، استرس­های روانی- اجتماعی مرتبط با کار و استرس­های روانشناختی مرتبط با کار را تكميل نمودند. نتايج به­صورت نسبت شانس خام و تطبيق شده و فاصله اطمينان 95% بيان گرديد. نتایج: 324 نفر (81%) پرستاران شرکت­کننده طی یک سال گذشته حداقل یک بار کمردرد را تجربه کرده­اند. میانگین سنی شرکت­کنندگان 2/6+ 4/32 سال، میانگین قد 7/8+7/166 سانتی­متر و میانگین وزن 8/9+7/67 کیلوگرم بود. از میان مشخصات فردی زن بودن (8/2OR=)، انجام ورزش منظم (4/0OR=)، افزایش قد به ازای هر سانتی­متر (06/1OR=) و افزایش وزن به ازای هرکیلوگرم (04/1OR=) ارتباط معناداری با کمردرد نشان دادند. از میان عوامل روانی- اجتماعی مرتبط با کار مشاهده شد به ازای هر یک درجه افزایش در شدت نارضایتی ارتباط با همکاران احتمال بروز کمردرد 80% بیش­تر می­شود و از میان عوامل استرس­زای شغلی به ازای هر یک درجه افزایش در طیف (همیشه، بیش­تر اوقات، بعضی وقت­ها، هرگز) عجله در کار احتمال بروز کمردرد 40% کم­تر می­شود. نتیجه­گیری: کمردرد یکی از جدی­ترین مشکلات شغل پرستاري می­باشد که با استرس­های روانشناختی محیط کار در ارتباط است. بنابراین تعدیل و اصلاح محیط روانی- اجتماعی بیمارستان و آموزش تکنیک­های کاهش استرس به­منظور پيشگیری یا کاهش عوارض ناشی از کمردرد ضروری به­نظر می­رسد

Title: بررسی شیوع لكه¬هاي مغولي و برخی عوامل مؤثر بر آن در نوزادان شهر تهران

http://knh.shmu.ac.ir/index.php/site/article/view/194

مقدمه: لكه‌هاي مغولي (Mongolian Spots- Congenital Dermal Melanocytosis) شايع­ترين ضايعات پيگمانته در نوزادان مي­باشند که به­صورت ماكول­هاي مادرزادي با اندازه و رنگ­هاي متنوع ديده مي‌شوند. شايع­ترين مكان لكه‌هاي مغولي در قسمت پایین کمر (ناحيه ساكروگلوتئال) مي‌باشد. شيوع اين لكه‌ها از 1% در نژاد قفقازی تا بیش از 90% در مغول­ها گزارش شده است. تاکنون مطالعات محدودي در مورد بررسي فراواني لكه‌هاي مغولي در ايران انجام گرديده است بنابراين مطالعه حاضر به بررسي شیوع اين لکه­ها و مکان­های آن در نوزادان بيمارستان لولاگر تهران مي­پردازد. مواد و روش­ها: اين مطالعه توصيفي- تحليلي به­مدت 2 سال، در فاصله سال­هاي 85-1383، در بيمارستان لولاگر تهران انجام پذيرفت. در اين فاصله 1706 نوزاد توسط پزشک متخصص اطفال از نظر وجود لكه­هاي مغولي و مكان­هاي شايع آن مورد بررسي قرار گرفتند. نتایج: بروز لكه­هاي مغولي در بيمارستان لولاگر تهران 3/37% (637 نوزاد) بود. شايع­ترين مكان لكه­هاي مغولي ناحيه ساكرال و بعد از آن ناحيه گلوتئال بود. بين وجود لكه مغولي با جنس، ترم و پره ترم بودن نوزاد، سن مادران، وزن نوزادان و نوع زايمان، ارتباط معنادار وجود نداشت (05/0<P). نتيجه­گيري: در بيمارستان لولاگر تهران میزان فراوانی لکه مغولی شبیه مطالعات قبلی انجام شده در شهرهای تهران و یزد می­باشد اما با مطالعات انجام شده در شهرهای اهواز و بوشهر متفاوت است. انجام يك مطالعه نژاد شناختي تكميلي بر روی نمونه­ای از نوزادان که معرف همه نوزادان کشور باشد ضروری به­نظر می­رسد.

Title: ارزیابی نقاط قوت و نقاط ضعف طرح پزشک خانواده

http://knh.shmu.ac.ir/index.php/site/article/view/195

مقدمه: با توجه به اجراي طرح پزشك خانواده در كشور هدف اين پژوهش ارزیابی نقاط ضعف و قوت طرح در شهرستان مراغه، مي­باشد. مواد و روش­ها: پژوهش حاضر یک مطالعه توصیفی و مقطعی بوده و جامعه آماری این پژوهش را جمعیت تحت پوشش، پزشکان خانواده، مدیران و کارکنان مراکز بهداشتی درمانی تشکیل می‏دادند. تعداد نمونه مدیران و کارکنان، برابر با کل جامعه آماری بود، و حجم نمونه‏ی جمعیت، 375 نفر بود. روش جمع‌آوری داده‏ها، پرسش­نامه و فرم جمع‌آوری داده‏ها بود که روایی و پایایی آن­ها قبل از شروع مطالعه مورد تأیید قرار گرفت. نتایج: حدود 97 درصد از جمعیت، از اجرای طرح پزشک خانواده مطلع بوده، 6/97 درصد آن­ها حداقل یک بار به پزشک خانواده مراجعه کرده بودند و 8/96 درصد آنان، دفترچه بیمه خدمات درمانی دریافت کرده بودند. نقاط قوت بارز طرح پزشک خانواده شامل: تشکیل پرونده سلامت، مراقبت بهتر و مؤثرتر از مادران باردار و کودکان زير 6 سال، دسترسی آسان روستاییان به پزشک و دارو، کاهش هزینه‏های درمان بود و از نقاط ضعف عمده آن، می­توان به مراجعات بیش از حد و ازدحام بیماران در خانه‌های بهداشت، عدم تأمین شغلی برای کارکنان، پرداخت نشدن به موقع حقوق و مزایای اعضای تیم سلامت، محدود بودن زمان دسترسی به پزشک خانواده در روستا اشاره نمود. نتیجه­گیری: براساس یافته­های این تحقیق پیشنهادهای زیر، به­منظور رفع نقاط ضعف و بهبود کیفیت خدمات و افزایش رضایت‌مندی بیماران، مطرح می‌شود: تشکیل پرونده سلامت الکترونیکی برای جمعیت تحت پوشش، ایجاد انگیزه و توجیه پزشکان متخصص در نحوه برخورد با بیماران ارجاعی، باز تعریف دقیق جمعیت تحت پوشش، حل مشکلات معیشتی اعضای تیم سلامت، و پرداخت به موقع حقوق و مزایای آنان.

Title: بررسي الگوي خواب و ميزان خواب¬آلودگي در جمعيت بالاي 15 سال شهر شاهرود

http://knh.shmu.ac.ir/index.php/site/article/view/196

مقدمه: با توجه به شيوع نسبتاً بالاي اختلالات خواب در جمعيت عمومي و تأثير آن بر عملكرد ذهني و جسمي افراد، اين مطالعه، با هدف بررسي الگوي خواب و خواب­آلودگي روزانه و عوامل مؤثر بر آن در جمعيت شهري شهر شاهرود انجام شده است. مواد و روش­ها: در اين مطالعه از تعداد 400 نفر نمونه انتخاب شده به روش تصادفي دو مرحله­اي تعداد 377 نفر به يك پرسش­نامه 48 سؤالي پاسخ دادند. به­منظور بررسي ميزان خواب­آلودگي از پرسش­نامه اپوورث و براي بررسي آپنه خواب و خرخر كردن از يك مقياس 4 درجه­اي استفاده شده است. نتايج: ميانگين سني افراد تحت مطالعه 30 سال مي­باشد و زنان 3/62% جمعيت تحت مطالعه را تشكيل مي­دادند. در اين مطالعه 41% نمونه­ها بر اساس معيار خواب­آلودگي اپوورث  نمره بالاتر از 6 كسب نموده و 9/13% آن­ها داراي خواب­آلودگي وسيع روزانه بودند (نمره بالاتر از 10). ميانگين نمره خواب­آلودگي برابر 2/6 با انحراف معيار 8/3 گزارش شده و 6/1% افراد بر اساس علايم مبتلا به آپنه خواب بودند. 12% نمونه­ها كم­تر از 6 ساعت در شبانه­روز مي­خوابيدند و ميانگين و ميانه زمان بيدار شدن از خواب، ساعت 7 صبح مي­باشد و بيش ار 40% افراد بعد از ساعت 12 شب مي­خوايند. نتيجه­گيري: با توجه به شيوع بالاي اختلالات خواب افزايش آگاهي افراد نسبت به اهميت مسأله خواب و پيامدهاي ناشي از آن ضروري مي­باشد.
